# Alt HealthWatch

**DOI:** 10.5195/jmla.2017.258

**Published:** 2017-10-01

**Authors:** Merete Christianson

Alt HealthWatch has evolved from a CD-ROM-based database in 1998 [1] to its present online iteration, which indexes complementary and alternative medicine (CAM) periodicals, often including full-text articles. As stated on its website, Alt HealthWatch’s purpose is to be a “resource for alternative and holistic approaches to health care and wellness” [2]. To that end, it succeeds. However, the focus of this review is the quality of Alt HealthWatch as a resource for patrons, rather than what it does and does not contain.

Little information is available on the database’s website as to who the main audience is. Given the mix of consumer and trade magazines with peer-reviewed journals, one can assume a broad mix of users as the audience: from CAM consumers to CAM practitioners to medical professionals with an interest or interested patients. It also covers a very broad range of CAM practices, such as Chinese medicine, natural products and supplements, chiropractic, acupuncture, mind-body medicine, yoga, and many more.

As an EBSCO product, Alt HealthWatch includes the features and functionality of all EBSCO databases, including a robust advanced search; publication searching; a variety of search modes, including Boolean or EBSCO’s SmartText Searching; and limiters such as source type, publication date, and language. Additionally, Alt HealthWatch offers controlled-vocabulary subject searching for precise results. Usability is generally high: the search box (or search boxes if using the advanced search) is familiar and relatively simple for users to understand. Results pages are not cluttered and are designed to allow users to easily delineate among title, citation information, subject terms, and other information, depending on whether they have chosen a brief or detailed results format. Full-text availability is easily assessed by whether or not a portable document format (PDF) or hypertext markup language (HTML) icon is displayed beneath each result, and the full text itself is only a click away.

One feature that comes in handy in Alt HealthWatch and other EBSCO databases is the ability to choose how results are sorted: by relevance, date newest, date oldest, source, or author. However, during the course of this review, the relevancy rankings of the searches and results being analyzed changed unexpectedly without any changes to search terms or strategies. While possibly a fluke or an update, it was nevertheless frustrating.

## CONTENT

Not all of the journals, magazines, and trade publications indexed in Alt HealthWatch meet the vendor’s stated claim of providing “Authoritative Content from Reputable Sources” [2]. The evaluation of this material would almost certainly differ depending on the background and views of the user. This review was approached from an evidence-based practice standpoint. Therefore, the content was reviewed with particular concern for whether claims were backed up by research evidence and how strong that evidence was: randomized controlled trials and reviews or case studies and editorials.

Two separate searches were conducted in Alt HealthWatch: “massage AND childbirth” and “vaccines and safety.” They were chosen for their relative simplicity—something an average user might try—as well as the fact that there has been evidence-based research conducted on both topics. Admittedly, they are not especially high-quality searches, so results were not expected to have a high degree of precision and relevance. Searches with subject terms as well as keywords are, of course, an option for the more experienced researcher.

### “Massage AND childbirth” search

In a Cochrane systematic review on the topic of pain management in labor, the authors concluded, “The limited data available suggest massage may be a helpful modality for pain management in labour; however, there is insufficient evidence to make clinical recommendations” [3]. Using that conclusion as a starting point, the first fifty results from a search for “massage AND childbirth” in Alt HealthWatch were analyzed for content, currency, and authority.

The results were a mix of sixteen academic journal articles and thirty-four articles from other types of periodicals. Ten of these fifty were from the *International Journal of Childbirth Education*, a peer-reviewed journal published by the International Childbirth Education Association. The information in this journal was of high quality, and one of the most relevant articles from it, “Natural Labor Pain Management” [4], cited the Cochrane review and presented a review of the current evidence base for massage with the caveat that “there was not enough evidence to support the effectiveness of massage or reflexology therapy.” Nurses, public health professionals, and educators were the main contributors to the journal. Additionally, all the articles from this journal in the first fifty relevancy-ranked results were from 2012 or later.

Most of the articles in the first fifty results were from a non-peer-reviewed publication, *Midwifery Today*: twenty-three of them, in fact. *Midwifery Today* bills itself as a publication for practicing midwives; as such, it included articles ranging from personal experiences to discussions of various midwifery practices—including CAM interventions—to summaries of new studies of interest. One particularly relevant article covered both why massage could be helpful during labor and different techniques. While it did not explicitly refer to the limited evidence base for massage, it stressed following and listening to the needs of the mothers, another central component of evidence-based practice. The articles from this magazine covered a time span of 2001 to 2017 in the first fifty results, so not quite current; however, the majority were from the 2010s. Additionally, many of the articles were written by practicing midwives.

Overall, the relevant articles in first fifty results on this topic in Alt HealthWatch were of decent quality and generally matched the evidence base. As noted before, the relevancy rankings changed in the middle of the process for an unknown reason, a frustrating event for this reviewer and possibly more so for other users.

One further issue arose when the results of this search were analyzed. One of the settings available through EBSCO is to automatically have search expanders applied when a search is performed, including the options to “Apply related words” and “Also search within the full text of the articles.” While some irrelevant results were expected since only two keywords were used, there were a surprising number of exceptionally off-topic articles in the top fifty relevancy-ranked results. One article about celebrity Maria Menounos was ranked as the tenth most relevant result, despite the fact that the word “massage” appeared only twice in the full text of the article and nowhere in the title, abstract, or subject terms. Turning off those expanders improved the relevance of the results.

### “Vaccines AND safety” search

While a source of some controversy, often among CAM practitioners [5], vaccines are widely acknowledged in the medical field to be safe and effective. As one Cochrane review states, “Existing evidence on the safety and effectiveness of MMR vaccine supports current policies of mass immunization aimed at global measles eradication and in order to reduce morbidity and mortality associated with mumps and rubella” [6]. Another review concludes, “Evidence was found for an association of several serious [adverse effects] with vaccines; however, these events were extremely rare: absolute risk is low” [7].

These evidence-based conclusions are not frequently represented in an Alt HealthWatch search for “vaccines AND safety.” The results are much more relevant than the “massage AND childbirth” search, with 45 of the first 50 results being relevant to the topic. However, 34 of those 45, or about 75% of the relevant articles, present views on vaccines that do not align with scientific evidence. In addition, the periodical that had the most results in the first 50 was the *Townsend Letter*, with 15 articles. With titles like “Voodoo Science: The Myth of Vaccine Efficacy” and “Vaccines’ Dark Inferno: What Is Not on Insert Labels?”, it is clear these articles do not agree with the evidence base but, moreover, use highly sensationalized writing to make their case.

The claim on the Alt HealthWatch website that the database includes information from “reputable sources” takes a hit in this case. The *Townsend Letter* explains on its own website, “We encourage reports which frequently are not data-based but anecdotal. Hence, information presented may not be proven or factually correct” [8].

Twelve of the first fifty articles were from the *American Journal of Homeopathic Medicine*. While billed as a peer-reviewed academic journal, it too engages in sensationalist writing, with articles such as “Vaxxed: From Cover-Up to Catastrophe: Parts I & II” (a positive review of the movie of the same name) and “Measles Madness: Parts I & II.” These also presented information that did not align with evidence-based medicine.

The eleven articles that did align with current scientific evidence came primarily from the peer-reviewed *Journal of the Canadian Chiropractic Association*, with three articles, and *FDA Consumer*, with five. Unfortunately, the articles from *FDA Consumer* span the years of 1995 to 2007, the year it ceased print publication, and so while they were evidence based, they were not particularly current.

Alt HealthWatch, in this search, did not prove to be a very useful resource. The majority of the first fifty articles did not agree with current medical evidence, and many of those that did were over ten years old. Additionally, the authors of the articles that took anti-vaccination stances had no expertise in the area and cited no scholars that were credentialed in vaccinology.

## CONCLUSION

Alt HealthWatch, indeed, fulfills its stated purpose: to be a “Resource for Alternative and Holistic Approaches to Health Care and Wellness.” However, CAM is a tricky subject:

It occupies a paradoxical position in modern medicine and healthcare: the plausibility and evidence base of many CAM treatments is very limited, and CAM approaches have been criticized and challenged by many scientists and physicians; despite this, some forms of CAM are popular among many lay people and a significant number of medical professionals. [9]

While many of the academic journals and magazines examined in this review make a point to cite or at least look to scientific evidence in their articles, a large number do not. Faculty working with academic librarians likely would not recommend this resource to students researching health topics, CAM or otherwise, as there are a number of databases, including PubMed, that do a better job of approaching the subject from an evidence-based perspective. Public or consumer health libraries may have more of a justification for including Alt HealthWatch in their collections, as it is an easy-to-use database on a topic that is popular, whether medical professionals like it or not.

For all resources, but particularly those on a rather controversial topic, it is not enough for librarians to simply include in them collections or decline to provide access. Resources like Alt HealthWatch remind us that we have a responsibility to go beyond providing information to guiding and instructing so that our users have the tools to think critically about the information that they find.
